# α-Synuclein Oligomers in Skin Biopsies Predict the Worsening of Cognitive Functions in Parkinson’s Disease: A Single-Center Longitudinal Cohort Study

**DOI:** 10.3390/ijms252212176

**Published:** 2024-11-13

**Authors:** Elena Contaldi, Milo Jarno Basellini, Samanta Mazzetti, Alessandra Maria Calogero, Aurora Colombo, Viviana Cereda, Gionata Innocenti, Valentina Ferri, Daniela Calandrella, Ioannis U. Isaias, Gianni Pezzoli, Graziella Cappelletti

**Affiliations:** 1 Parkinson Institute of Milan, ASST G. Pini-CTO, 20126 Milan, Italy; auroracol@hotmail.it (A.C.); viviana.cereda@gmail.com (V.C.); gionata.innocenti@asst-pini-cto.it (G.I.); ferri.v@virgilio.it (V.F.); calandrella@parkinson.it (D.C.); ioannis.isaias@asst-pini-cto.it (I.U.I.); gianni.pezzoli@gmail.com (G.P.); 2 Department of Biosciences, Università degli Studi di Milano, 20133 Milan, Italy; milo.basellini@unimi.it (M.J.B.); samanta.mazzetti@gmail.com (S.M.); alessandra.calogero@unimi.it (A.M.C.); graziella.cappelletti@unimi.it (G.C.); 3 Fondazione Grigioni per il Morbo di Parkinson, 20125 Milan, Italy; 4 Department of Neurology, University Hospital of Würzburg and Julius-Maximilian-University of Würzburg, 97080 Würzburg, Germany

**Keywords:** α-synuclein oligomers, skin biopsy, cognitive decline, Parkinson’s disease

## Abstract

α-synuclein oligomers within synaptic terminals of autonomic fibers of the skin reliably discriminate Parkinson’s disease (PD) patients from healthy controls. Nonetheless, the prognostic role of oligomers for disease progression is unknown. We explored whether α-synuclein oligomers evaluated as proximity ligation assay (PLA) score may predict the worsening of cognitive functions in patients with Parkinson’s disease. Thirty-four patients with PD and thirty-four healthy controls (HC), matched 1:1 for age and sex, were enrolled. Patients with PD underwent baseline skin biopsy and an assessment of cognitive domains including Mini-Mental State Examination (MMSE), Montreal Cognitive Assessment (MoCA), Clock Drawing Test, and Frontal Assessment Battery. At the last follow-up visit available, patients were either cognitively stable (PD-CS) or cognitively deteriorated (PD-CD). α-synuclein oligomers were quantified as PLA scores. Differences between groups were assessed, controlling for potential confounders. The relationship between skin biopsy measures and cognitive changes was explored using correlation and multivariable regression analyses. The discrimination power of the PLA score was assessed via ROC curve. To elucidate the relationship between skin biopsy and longitudinal cognitive measures, we conducted multivariable regression analyses using delta scores of cognitive tests (Δ) as dependent variables. We found that PD-CD had higher baseline PLA scores than PD-CS (*p* = 0.0003), and they were correctly identified in the ROC curve analysis (AUC = 0.872, *p* = 0.0003). Furthermore, ANCOVA analysis with Bonferroni correction, considering all groups (PD-CS, PD-CD, and HC), showed significant differences between PD-CS and PD-CD (*p* = 0.003), PD-CS and HC (*p* = 0.002), and PD-CD and HC (*p* < 0.001). In the regression model using ΔMMSE as the dependent variable, the PLA score was found to be a significant predictor (β = −0.441, *p* = 0.016). Similar results were observed when evaluating the model with ΔMoCA (β = −0.378, *p* = 0.042). In conclusion, patients with Parkinson’s disease with higher α-synuclein burden in the peripheral nervous system may be more susceptible to cognitive decline.

## 1. Introduction

Parkinson’s disease (PD) is one of the most common neurodegenerative disorders. The clinical picture is characterized by a wide array of motor and non-motor symptoms. Among them, cognitive dysfunction is one of the most debilitating symptoms as it significantly impacts the quality of life of patients and their caregivers; approximately 20% of de novo patients have mild cognitive impairment (MCI), and more than 40% of patients with normal cognition at baseline develop MCI within 6 years from motor symptoms onset [1]. Furthermore, more than 80% of subjects develop Parkinson’s disease dementia (PDD) 20 years after diagnosis [2]. The development of dementia is associated with poor prognosis as admission to residential care facilities or death may occur 3 to 4 years after dementia onset. 

The identification of patients with Parkinson’s disease at risk of cognitive decline is crucial in the management of the disease. In this regard, several candidate biomarkers have been proven to facilitate early diagnosis and predict cognitive decline in PD. The validation of such biomarkers has been developed around the elucidation of causative factors involved in cognitive decline, i.e., genetic, neuropathological, and inflammatory mediators [3,4,5]. Being the hallmark of the disease, α-synuclein proteinopathy represents one of the most widely explored avenues of research in the field of PD-related cognitive decline. Even though α-synuclein levels in the cerebrospinal fluid (CSF) of patients with PD are lower than in that of healthy controls, a meta-analysis failed to find any difference between patients with and without dementia [6]. Another study [7] reported significantly higher levels of α-synuclein oligomers in the CSF of patients with PDD and dementia with Lewy bodies (DLB) compared to patients with Alzheimer’s disease (AD). Additionally, several studies examining the role of amyloid-β 1–42 (Aβ42) reported the association between lower CSF levels of Aβ42 and worse cognition in PD [8]. More recently, seed amplification assays have been employed to reveal disease-specific misfolded α-synuclein aggregates, and a more prominent α-synuclein seeding kinetic profile has been associated with a faster cognitive decline in PD [9] and other Lewy body disorders [10]. Alongside the CSF, the peripheral nervous system could represent a valuable source for discovering novel biomarkers and pathogenic mechanisms involved in PD [11]. We recently reported the presence of α-synuclein oligomers within synaptic terminals of autonomic fibers of the skin and demonstrated that these aggregates may reliably discriminate patients with PD from healthy controls (HC) [12]. Nonetheless, their role in the progression of PD is still unknown. In the present work, we focused on a cohort of patients with Parkinson’s disease prospectively followed up to evaluate whether peripheral α-synuclein oligomers accumulation may predict the future worsening of cognitive functions. 

## 2. Results

A total of 34 patients with PD (55.8% males) and 34 HC (average age at skin biopsy = 60 ± 11.1 years, 55.8% males) were enrolled in the present study. In the PD group, the age at skin biopsy was 59.76 ± 8.32 years, and the average disease duration was 5.21 ± 3.79 years. A total of 10 (29.41%) patients displayed pRBD, 18 (52.94%) constipation, 9 (26.47%) reported hyposmia, and 6 (17.6%) had neurogenic orthostatic hypotension. The Mean Unified Parkinson’s Disease Rating Scale (UPDRS) part III was 19.64 ± 12.56, and most patients had Hoehn and Yahr (HY) staging between 1 and 2.5 (see Table 1 for complete demographic and clinical data details).

At baseline, 8/34 (23.5%) were classified as PD-MCI and 26/34 (76.5%) as PD with normal cognition (PD-NC). After an average follow-up of 5.03 ± 1.64 years, 13/34 patients (38.2%) were cognitively deteriorated (PD-CD): 5/13 (38.5%) converted into PD-MCI and 8/13 (61.5%) into PDD. The remaining 21/34 (61.8%) were cognitively stable (PD-CS). PD-CS were similar to PD-CD in terms of baseline clinical and demographic characteristics. However, PD-CD displayed lower scores than PD-CS in the Mini-Mental State Examination (MMSE; *p* = 0.001), Montreal Cognitive Assessment (MoCA; *p* < 0.001), and Clock Drawing Test (CDT; *p* = 0.012), and had a higher prevalence of probable REM sleep behavior disorder (pRBD; *p* = 0.022) and MCI (*p* = 0.002). No significant differences were detected between patients with PD and HC regarding age and sex (respectively, *p* = 0.931, *p* = 1.0). The percentage changes of cognitive tests between baseline and follow-up examinations in PD-CS and PD-CD are shown in Supplementary Table S1. 

**Figure 1:**
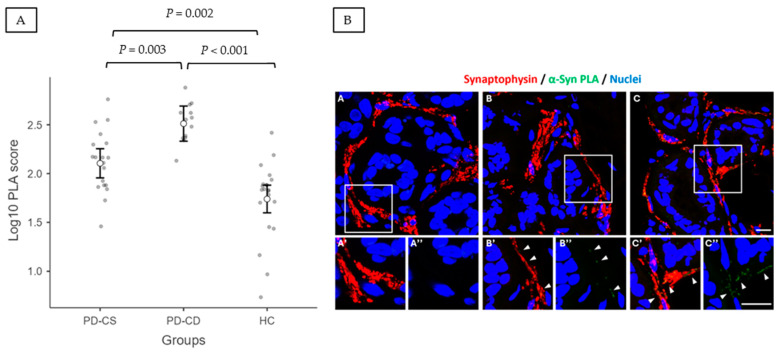
(**A**) Estimated marginal means (ANCOVA analysis adjusting for age and disease duration) for PLA score by groups. Error bars represent the 95% confidence interval. (**B**) Representative images of α-synuclein oligomers, detected by PLA (green signal), within sudomotor synaptic terminals (red signal) of HC (**A**–**A”**), PD-CS (**B**–**B”**), and PD-CD (**C**–**C”**). Insets in (**A’**,**A”**), (**B’**,**B”**), and (**C’**,**C”**) show 2× magnified images of the squared area in (**A**–**C**), respectively. Nuclei are counterstained in blue. Arrowheads = PLA positive signal. Scale bar: 10 μm.

**Figure 2:**
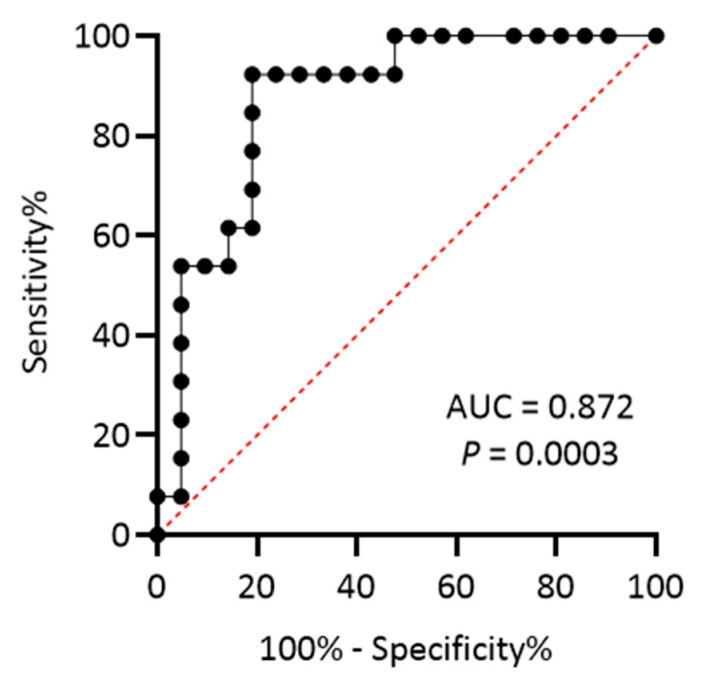
ROC curve analysis of PLA score to discriminate between PD-CS and PD-CD.

With regard to skin biopsy measures, patients with PD displayed higher PLA scores than HC (233.55 ± 179.75 vs. 45.42 ± 56.76; *p* < 0.0001). Additionally, PD-CD had higher baseline PLA scores than PD-CS (363.68 ± 167.50 vs. 152.99 ± 136.58, *p* = 0.0003). This result was confirmed after correcting for potential interfering factors (age, sex, and disease duration) in the analysis of covariance (ANCOVA; adjusted *p* = 0.00007). ANCOVA analysis with Bonferroni correction considering all groups (PD-CS, PD-CD, and HC) showed significant differences between PD-CS and PD-CD (adjusted *p* = 0.003), PD-CS and HC (adjusted *p* = 0.002), and PD-CD and HC (adjusted *p* < 0.001) (see Figure 1A,B). We then used a receiver operating characteristic (ROC) curve to determine whether the PLA score could reliably identify PD-CD subjects and found an area under the curve (AUC) = 0.872 (95% CI 0.751–0.993), *p* = 0.0003 (see Figure 2). 

According to Youden’s index, a cut-off point ≥ 211.97 showed 92.31% sensitivity and 81% specificity. Significant correlations were found between baseline cognitive measures and the PLA score but not synaptic density values. In more detail, the PLA score showed an inverse correlation with MMSE (r = −0.476, *p* = 0.006) and MoCA (r = −0.440, *p* = 0.012). Additionally, significant correlations were found with delta scores (Δ) of MMSE and MoCA (respectively, r = −0.395, *p* = 0.025; r = −0.363, *p* = 0.041). There was no significant relationship with other clinical–demographic measures. 

**Figure 3:**
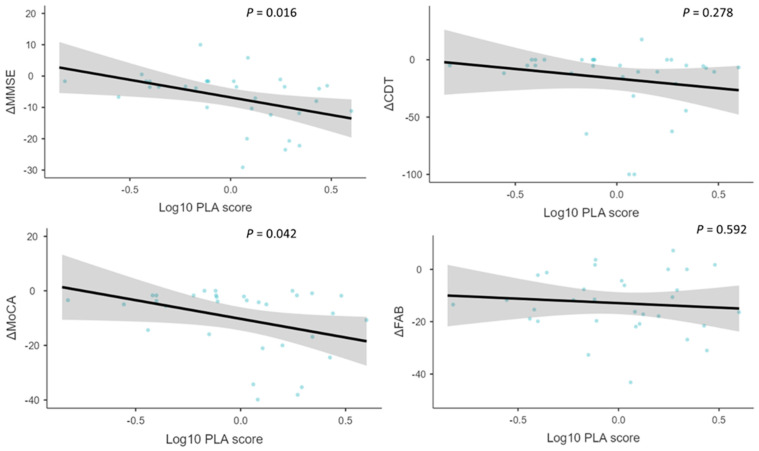
Linear regression analysis using PLA score as the independent variable and cognitive test changes as dependent variables. Models include age and disease duration as independent variables. Grey-shaded areas around each regression line represent the 95% confidence intervals.

To further elucidate the relationship between skin biopsy and longitudinal cognitive changes, we conducted multivariable regression analyses using Δ of cognitive tests as dependent variables. Non-collinear independent variables were age, disease duration, and log-transformed PLA score. In the model using ΔMMSE as the dependent variable, we found that the PLA score was a significant predictor (β = −0.441, *p* = 0.016; R_2_ = 0.219). Similar results were observed when evaluating the model with ΔMoCA as the dependent variable (β = −0.378, *p* = 0.042; R_2_ = 0.167). Moving to other cognitive measures, no statistically significant result was found for the PLA score when considering the dependent variables ΔCDT (*p* = 0.278) and ΔFAB (*p* = 0.592) (see Figure 3). We further considered separate models, including baseline cognitive status (PD-MCI vs. PD-NC) and PLA score, as independent variables, and the latter was confirmed as a significant predictor of ΔMMSE (*p* = 0.027), whereas a non-significant trend toward significance (*p* = 0.1) was found for ΔMoCA.

After stratifying our population based on PLA score (PLA_high_ ≥ 211.97; N = 16, PLA_low_ < 211.97; N = 18), we did not find statistically significant differences regarding age at skin biopsy, age at PD onset, sex, disease duration, UPDRS-III scores, HY stage, motor phenotype, levodopa equivalent daily dose (LEDD), presence of hyposmia, constipation, and dysautonomia. However, patients in the PLA_low_ subgroup were mostly pRBD− (83.3%), whereas only 16.7% were pRBD+ (Fisher’s exact test *p* = 0.088). 

## 3. Discussion

This study reports different amounts of α-synuclein oligomeric deposits in the skin of patients with Parkinson’s disease with distinct longitudinal changes in cognitive functions. In more detail, we found that (i) PD-CD have higher baseline PLA scores than PD-CS, and this score adequately discriminates PD-CD; (ii) there is a significant association between PLA score and longitudinal changes in MMSE and MoCA test scores. 

These results align with the current knowledge of the role of α-synuclein oligomers in PD pathology. Oligomers are small, soluble protein aggregates with peculiar functional properties and are regarded as an intermediary between soluble monomeric proteins and insoluble mature fibrils [13]. Indeed, recent studies have shown that soluble oligomers are toxic species that induce neuronal damage in several neurodegenerative disorders [11,14]. Neurodegeneration driven by α-synuclein oligomers might be caused by their interaction with many cellular partners, including membranes and cytoskeleton [15,16]. Furthermore, α-synuclein oligomers induced an inflammatory response in microglia through the activation of Toll-like receptor 1 and 2 pathways [17,18], whereas the monomeric and fibrillary forms of α-synuclein did not [19,20]. Additionally, α-synuclein oligomers determine synaptic dysfunction through a Ca^2+^-dependent release of glutamate from astrocytes, leading to a chronic increase in glutamate, thus activating neuronal extrasynaptic NMDA receptors (eNMDARs) and contributing to synaptic damage [21]. Diógenes et al. also showed that α-synuclein oligomers increase basal synaptic transmission through NMDA receptor activation and impair long-term potentiation in rat hippocampus [22]. 

With regard to human studies, the contribution of α-synuclein oligomers to motor and non-motor progression of PD remains elusive. Sekiya and colleagues [23] described the distribution of α-synuclein oligomers in different cortical and subcortical regions of patients with PD using the PLA technique. α-synuclein oligomers’ burden was significantly greater in the neocortex, while Lewy-related pathology (LRP, i.e., Lewy bodies and Lewy neurites) was greater in subcortical regions, including the brainstem. The authors observed abundant α-synuclein oligomers in the hippocampus of patients with cognitive impairment, whereas this association was not observed with LRP. These findings suggest a widespread distribution of α-synuclein oligomers early in the disease process and their contribution to cognitive impairment in PD. 

However, the prognostic value of α-synuclein oligomers has remained unclear due to the heterogeneity of laboratory testing and differences in sample collection from tissues/fluids [24]. A study investigating the role of CSF biomarkers in a cohort of 94 de novo patients with PD [25] observed that CSF oligomeric-α-synuclein longitudinally increased over the 4-year follow-up and correlated with PD motor progression. Such an association was not observed with cognitive aspects, probably because patients were cognitively intact at baseline, with no significant changes at follow-up evaluations, and only the MMSE score was used to track cognitive impairment.

Concerning skin biopsies, in our previous cross-sectional study involving idiopathic and monozygotic twin patients with PD [10], we did not find any significant association between PLA score and clinical features, including disease duration and the severity of motor symptoms. To elucidate the prognostic capacity of α-synuclein-PLA, a longitudinal study by Vacchi et al. [26] evaluated skin biopsies of patients with PD, atypical Parkinson’s disease, and HC. After a 24-month follow-up, a progression of denervation was observed, and baseline intraepidermal nerve fiber density (IENFD), but not α-synuclein-PLA, was associated with greater cognitive and motor decline. Notably, we report an association between PLA score and the worsening of cognitive functions evaluated through the MMSE and MoCA tests. Some methodological differences can explain such discrepancies: (i) in our study, skin biopsies were collected from the volar forearm, whereas Vacchi et al. performed their analysis from cervical and ankle sites, plus the selection of different inclusion techniques (frozen vs. paraffin-embedding); (ii) our α-synuclein-PLA assay exploited a polyclonal antibody directed toward a different epitope; (iii) we quantified oligomers deposition co-localized within synaptophysin-positive structures and not protein gene product (PGP)9.5; (iv) our cohort of patients with PD was prospectively assessed over a longer period. 

In line with our results, a recent cross-sectional study [27], evaluating cutaneous α-synuclein deposition in a large cohort of patients with PD and other synucleinopathies, reported significant correlations between phosphorylated α-synuclein and MoCA score (r = −0.32, *p* < 0.001). Intriguingly, significant correlations were also found with the Orthostatic Hypotension Questionnaire score and the RBD screening questionnaire score, reinforcing the concept of higher peripheral α-synuclein burden in the body-first subtype of PD [28]. In this regard, we similarly report that PLA_low_ patients are mostly pRBD-, whereas the lack of tailored questionnaires and instrumental assessment of autonomic symptoms may have resulted in an underestimation of the relationship between PLA score and dysautonomia. Another study [29] also reported high α-synuclein seeding activity in real-time quaking-induced conversion assay (RT-QuIC) in skin biopsy samples of patients with PD with RBD, constipation, and cognitive impairment. Taken together, these results suggest the correlation between skin α-synuclein pathology and both “central” and “peripheral” symptoms, thus supporting the systemic nature of α-synuclein deposition.

Several limitations of the present research should be highlighted: (i) the small sample size, which limited the power of statistical analyses; (ii) the application of level I abbreviated assessment for the diagnosis of cognitive impairment, which prevented the further classification of MCI in single or multiple domains; (iii) the inclusion of intermediate-stage patients with PD with a prevalence of MCI at baseline (23.5%) in line with previous studies [30,31], which could have determined a selection bias and influenced baseline correlations between PLA score and cognitive measures; (iv) the lack of other imaging or biological biomarkers, which were not a requirement for study enrollment. Concerning these limitations, (i) despite the limited sample size, relevant covariates such as age and disease duration were taken into careful consideration in ANCOVA and multivariable regression models; (ii) since comprehensive neuropsychological testing is not always possible because of time, cost, or patients’ inability to cooperate with a long assessment, the application of abbreviated level I PD-MCI criteria in clinical and research settings has been explored in several studies. For instance, an assessment with at least one test for each of the five cognitive domains independently contributed to the hazard of PDD [32]. Similarly, Boel et al. [33] found that MMSE and MoCA predicted the conversion to PDD and level I neuropsychological assessment showed good diagnostic accuracy (86%); (iii) we could not enroll a sufficient number of early-stage patients with PD due to the selection bias in our Parkinson Center (tertiary referral hospital for PD). However, meaningful information regarding distinct cognitive trajectories was provided by including patients with at least 3 years of consecutive neuropsychological assessments. 

## 4. Materials and Methods

### 4.1. Study Population

This longitudinal study involves a cohort of patients with PD enrolled at the Parkinson Institute of Milan (Italy) by neurologists experienced in movement disorders and participating to the Biobank of the Parkinson Institute [34]. Study procedures were approved by the local Ethics Committee (protocol 3499/23), and this research was carried out in agreement with the principles of the Declaration of Helsinki and later amendments. All patients gave their written informed consent. 

Currently, the Biobank includes 360 skin biopsies collected from 283 patients and 77 HC. Patients were not considered eligible for the study if they had a history of diseases or other conditions potentially leading to peripheral neuropathy (i.e., diabetes mellitus) or general contraindications for performing a skin biopsy. For the present investigation, we excluded the following: (i) patients with atypical parkinsonism (n = 52); (ii) patients with genetic forms of PD (n = 78); and (iii) other movement disorders (n = 22). Among the 152 patients with a diagnosis of idiopathic PD according to the UK Brain Bank criteria [35], we further excluded those with less than 3 years of follow-up after skin biopsy assessment (m = 85) and those with a diagnosis of PDD at the time of skin biopsy (N = 46) [36]. 

The analyzed cohort consisted of 34 patients with Parkinson’s disease enrolled from January 2015 to December 2019. Skin biopsies from 34 HC were carefully selected to be age- and sex-matched to PD subjects (1:1). Parkinsonian patients underwent at baseline an abbreviated assessment (level I) of cognitive domains according to the Movement Disorder Society (MDS) Task Force [37] and complete motor evaluation in medication “on” condition (UPDRS-III and HY scale [38,39]). In more detail, cognitive assessment included MMSE [40], CDT [41], MoCA [42], and frontal assessment battery (FAB [43]). We used Italian-validated versions, and raw scores were adjusted for age, sex, and educational level according to established correction grids. At baseline, patients were classified as PD-NC or PD-MCI. Cognitive tests were collected every year; at the last follow-up visit available, patients with PD were either PD-CS if PD-NC/PD-MCI were classified in the same group as baseline, or cognitively deteriorated if PD-NC converted into PD-MCI or PD-MCI converted into PDD [34,35]. Changes in cognitive tests were calculated as follows: [(test score_follow-up_ − test score_baseline_)/test score_baseline_] × 100. 

Other relevant data were collected: LEDD (according to [44,45]); the presence of hyposmia according to medical reports; pRBD according to a cut-off score ≥ 6 in the RBD Screening Questionnaire (RBDSQ) [46]; constipation (following Rome III criteria [47]); neurogenic orthostatic hypotension (based on specific prescription patterns and/or the detection during clinical examination of a fall of at least 20 mmHg in systolic blood pressure (30 mmHg in patients with supine hypertension) and/or 10 mmHg in diastolic blood pressure within 3 min of standing [48]); and motor subtype [49]. 

### 4.2. Skin Biopsy

The procedure has been extensively described elsewhere [10]. Briefly, skin biopsies obtained from the volar forearm were fixed in Zamboni solution for 24 h at 4 °C, embedded in paraffin, and cut in 3 µm thick serial sections using a microtome (MR2258, Histo-Line Laboratories, Milan, Italy). Then, for each sample: (i) one section was stained with hematoxylin and eosin to verify the correct morphology and to check the presence of sweat glands; (ii) three sections were used to perform the proximity ligation assay (PLA) technique to localize α-synuclein oligomers, together with classical immunofluorescence staining for synaptophysin to highlight autonomic synaptic terminals. Experiments were performed, according to the manufacturer’s instructions, using a Duolink kit (Sigma-Aldrich, St. Louis, MI, USA), as previously described [10]; briefly, anti-α-synuclein antibody (clone S3062, rabbit polyclonal, Sigma-Aldrich, St. Louis, MI, USA) was conjugated overnight to either PLUS or MINUS probes and stored at 4 °C. 

First, sections underwent deparaffinization and rehydration; then, antigen retrieval was performed with 10% formic acid (10 min). After a 20 min saturation step with 1% bovine serum albumin (BSA) in 0.01 M phosphate-buffered saline (PBS) plus 0.1% Triton X-100, sections were incubated with α-synuclein-PLUS and α-synuclein-MINUS probes (1:100 in PLA diluent) and mouse anti-synaptophysin (clone DakSynap, 1:100; Agilent, Santa Clara, CA, USA) for 2 h at 37 °C. Then, the amplification reaction was obtained, and sections were incubated with the secondary antibody AlexaFluor^TM^ 568 donkey anti-mouse (Invitrogen, Waltham, MA, USA). Finally, nuclei were stained with Hoechst 33342 (1:1000; Life Technologies, Waltham, MA, USA) for 10 min and mounted using Mowiol-DABCO. Images were collected at a Nikon spinning disk confocal microscope using an oil-immersion 60× objective and analyzed with ImageJ software version 1.54. 

PLA score was calculated as follows [10]: the area of PLA signal within synaptic terminals (synaptophysin-positive) was normalized for synaptic density (synaptophysin-positive area/total area of the sweat gland). This measure provides a quantification of α-synuclein oligomers within synaptic terminals of skin autonomic fibers.

### 4.3. Statistical Analyses

This is a hypothesis-generating longitudinal study. We considered the correlation between the change in cognitive test scores and baseline α-synuclein oligomers as the primary outcome. Assuming a correlation coefficient r = 0.5, a two-tailed type I error = 5%, and power = 80%, we estimated a sample size of at least 28 patients (“pwr” package, RStudio 2022.07.2 + 576). Variables were expressed as counts (percentages) when categorical and as mean (standard deviation, SD) or median (interquartile range, IQR) when continuous according to data distribution. The normality of the data was assessed using the Shapiro–Wilk test. 

Differences between groups were analyzed by independent samples *t*-test or the non-parametric equivalent Mann–Whitney test as appropriate. ANCOVA was used to adjust for relevant covariates. ROC curve analysis was performed to explore biomarkers’ discriminatory power, obtaining the AUC and significance values. AUC interpretation was determined according to Mandrekar et al. [50]. The relationship between PLA score and cognitive assessment was investigated using Pearson’s or Spearman’s correlation analysis as appropriate. 

Finally, non-collinear independent variables associated with clinical symptoms were investigated using multivariable linear regression analysis (continuous variables on either a normal or log-transformed scale). Relevant independent variables were chosen based on univariate analysis and background literature [51,52]. All tests were two-tailed, and the significance level was *p* < 0.05. Analyses were performed using SPSS version 25 (IBM Corporation, Armonk, NY, USA), RStudio 2022.07.2 + 576, and Jamovi (Version 2.3.28). 

## 5. Conclusions

To the best of our knowledge, this is the longest longitudinal study (mean duration of follow-up = 5 years) evaluating α-synuclein oligomers from skin biopsies as feasible biomarkers to track cognitive changes in PD. In line with the hypothesis of a body-first phenotype, patients with higher α-synuclein burden in the peripheral nervous system may be more susceptible to cognitive decline. It will be crucial to determine the prognostic capacity of α-synuclein oligomers in larger cohorts of early-stage patients with PD to fulfill the currently unmet need for a reliable biomarker in the context of novel disease-modifying therapies, as well as to inform future strategies for more precise patient stratification.

## Figures and Tables

**Table 1:** Clinical and demographic data of our PD cohort and in cognitively stable (PD-CS) and cognitively deteriorated (PD-CD) subgroups.

Variables	PD Cohort (*n* = 34)	PD-CS (*n* = 21)	PD-CD (*n* = 13)	*p*-Value PD-CS vs. PD-CD
Age at skin biopsy, years, mean (SD)	59.76 (8.32)	59.67 (7.54)	59.92 (9.78)	0.932
Sex (male), n (%)	19 (55.9%)	12 (57.1%)	7 (53.8%)	1.0
Scholarity, years, mean (SD)	10.97 (3.69)	11.38 (4.09)	10.31 (2.98)	0.419
Age at PD onset, years, mean (SD)	54.56 (8.95)	54.48 (8.76)	54.69 (9.61)	0.709
Disease duration, years, mean (SD)	5.21 (3.79)	5.19 (4.11)	5.23 (3.37)	0.977
LEDD, mg/day, mean (SD)	616.55 (610.36)	626.91 (640.13)	603.89 (610.13)	0.761
HY scale (on) 1–2.5, n (%) 3, n (%)	32 (94.1%) 2 (5.9%)	20 (95.2%) 1 (4.8%)	12 (92.3%) 1 (7.7%)	1.0
UPDRS III score (on), mean (SD)	19.64 (12.56)	14.89 (7.87)	28.20 (15.69)	0.134
Tremor dominant phenotype, n (%)	10 (29.4%)	8 (38.1%)	2 (15.4%)	0.251
pRBD, n (%)	10 (29.4%)	3 (14.3%)	7 (53.8%)	0.022
Hyposmia, n (%)	9 (26.5%)	7 (33.3%)	2 (15.4%)	0.675
Constipation, n (%)	19 (55.9%)	11 (52.4%)	8 (61.5%)	0.630
Neurogenic orthostatic hypotension, n (%)	6 (17.6%)	3 (14.3%)	3 (23.1%)	0.653
Follow-up duration, years, mean (SD)	5.03 (1.64)	4.71 (1.65)	5.54 (1.56)	0.140
PLA score, mean (SD)	233.55 (179.75)	152.99 (136.58)	363.68 (167.50)	0.0003
Synaptic density, mean (SD)	0.019 (0.01)	0.0198 (0.010)	0.0195 (0.011)	0.906
MMSE score baseline, mean (SD)	28.07 (1.66)	28.87 (0.99)	26.78 (1.75)	0.001
CDT score baseline, mean (SD)	9.03 (1.23)	9.36 (1.04)	8.50 (1.37)	0.012
MoCA score baseline, mean (SD)	25.97 (1.32)	28.05 (1.93)	22.61 (1.04)	<0.001
FAB score baseline, mean (SD)	15.07 (1.57)	15.37 (1.51)	14.58 (1.61)	0.159
PD-MCI, n (%)	8 (23.5%)	1 (4.8%)	7 (53.8%)	0.002
Hypertension, n (%)	8 (23.5%)	6 (28.6%)	2 (15.8%)	0.444

Abbreviations: LEDD, levodopa equivalent daily dose; HY, Hoehn and Yahr scale; UPDRS, Unified Parkinson’s Disease Rating Scale; pRBD, probable REM sleep behavior disorder; PLA, proximity ligation assay; MMSE, Mini-Mental State Examination; CDT, Clock Drawing Test; MoCA, Montreal Cognitive Assessment; FAB, Frontal Assessment Battery; PD-MCI, Parkinson’s disease with mild cognitive impairment.

## Data Availability

Source data used for analyses presented in this study are available from the authors upon reasonable request.

## Supplementary Materials

The following supporting information can be downloaded at https://www.mdpi.com/article/10.3390/ijms252212176/s1.
