# Supplementary material for: α-Synuclein Oligomers in Skin Biopsies Predict the Worsening of Cognitive Functions in Parkinson’s Disease: A Single-Center Longitudinal Cohort Study
Source: Int J Mol Sci. 2024 Nov 13;25(22):12176. doi: 10.3390/ijms252212176 (PMC11594322; doi:10.3390/ijms252212176)
Supplement: Supplementary file 1 [file ijms-25-12176-s001.zip › ijms-3300938-supplementary.pdf]

Supplementary Table S1. Percentage changes of cognitive tests between baseline and follow-up examination in PD-CS and PD-CD subgroups. Negative delta values indicate worsening cognitive functions and are expressed as median (interquartile range). Between-group differences were assessed using the Mann-Whitney test.

**Change from baseline of cognitive tests (%)**	**PD-CS (*n* = 21)**	**PD-CD (*n* = 13)**	***P*-value PD-CS vs PD-CD**
MMSE	-1.72 (-3.5; -1.62)	-11.87 (-21.46; -6.01)	**0.001**
CDT	0 (-5.13; 0)	-21.05 (-63.60; -6.90)	**<0.0001**
MoCA	-2.10 (-4.44; -1.68)	-20 (-34.79; -9.52)	**<0.0001**
FAB	-7.64 (-14.44; -0.62)	-20.85 (-28.93; -12.09)	**0.004**

Abbreviations: MMSE, Mini-Mental State Examination; CDT, Clock Drawing Test; MoCA, Montreal Cognitive Assessment; FAB, Frontal Assessment Battery
